# Aneurysm at junction of inferior pancreaticoduodenal arcade in the setting of distal collateralization from proximally ligated splenic artery

**DOI:** 10.1016/j.radcr.2026.10.010

**Published:** 2026-10-31

**Authors:** Sayyed M.W. Hussain, Brian M. Currie

**Affiliations:** a St. Luke’s University Health Network, 1872 St. Luke's Boulevard, Easton, PA, 18045, USA; b Geisinger Medical Center, 1000 East Mountain Boulevard, Wilkes Barre, PA, 18711, USA

**Keywords:** Inferior pancreaticoduodenal artery, Aneurysm, Visceral, Embolization, Transplant, Hepatic congestion

## Abstract

Visceral artery aneurysms, particularly aneurysms involving the pancreaticoduodenal arteries, are exceptionally rare and they carry the potential to be unpredictably lethal. Typically, true aneurysms involving the pancreaticoduodenal arcades occur secondary to steno-occlusion of the celiac axis. However, here we present a case of an inferior pancreaticoduodenal arcade (IPDA) junctional aneurysm secondary to chronically elevated collateral arterial blood flow from the pancreaticoduodenal arteries to the splenic artery. The patient had massive splenomegaly almost a decade after liver transplant and splenic artery ligation. There are no similar reports in the literature, and this case supports the hypothesis that chronically elevated blood flow through the small-caliber arteries predisposes to aneurysm formation.

## Background

Aneurysms of visceral arteries have an accepted prevalence of 0.2% within the general population [1], with the incidence of superior mesenteric artery (SMA) aneurysm previously reported as 1 in 12000 consecutive autopsies [2]. Only 2% of all visceral artery aneurysms occur in pancreatic vessels [3], with the first known case of pancreaticoduodenal artery aneurysm (PDAA) reported in 1895 [4]. Since then, over 34 cases of PDAAs had been reported by 1993 [5] and at least a total of 125 cases by 2017 [6]. Whether due to improvements in imaging techniques alone or in conjunction with a combination of other factors, there has been an increase in the number of reported PDAAs [7]. A substantial fraction of these patients has been asymptomatic on presentation with the PDAA only incidentally diagnosed [1]. Others have presented more classically with vague abdominal pain or mesenteric angina symptoms, severe abdominal distension with signs of hematoma, and even in shock with hemodynamic collapse following rupture of the aneurysm [1]. A single instance of a patient presenting with signs and symptoms of obstructive jaundice secondary to an inferior pancreaticoduodenal artery (PDA) aneurysm is also noted in the literature [8]. Regardless, endovascular embolization of PDAAs is increasingly the treatment of choice in non-emergent settings given the high technical success rate and low risk of complications. This is particularly true when compared to the high risks associated with non-treatment and rupture—regardless of presenting symptoms and aneurysm size [1,7,9,10].

The significant majority of true PDAAs reported thus far, in the absence of connective tissue disorders, fall under the umbrella of Sutton-Kadir syndrome (steno-occlusion of the celiac axis) or a complication of compression of the celiac trunk (Dunbar Syndrome). Here we present a rare case of an IPDA junctional aneurysm in a liver transplant patient following splenic artery ligation and subsequent development of splenomegaly.

## Case

A 32-year-old physically-active male presented for work-up of suspected portal hypertension after a recent episode of esophageal variceal bleeding that required esophagogastroduodenoscopy (EGD) and banding at an outside hospital (OSH).

His past medical and surgical history was notable for glycogen storage disease Type 1A (von Gierke disease) for which he was treated with remote living-donor liver transplant. He initially required multiple OR take-backs over the first post-operative year and ultimately required splenic artery ligation for flow diversion to the graft. Several years later, he was also treated for hepatic vein stenosis and subsequent transjugular pressures showed no evidence of flow-limiting stenosis.

The leading hypotheses were recurrent venous outflow obstruction causing portal hypertension or increased portal inflow from massive splenomegaly (Fig. 1A) given his history of venous stenosis and unrevealing cross sectional imaging. A TIPS was not considered a viable option due to poor outcomes in post-transplant patients and no evidence of cirrhosis.

**Fig. 1:**
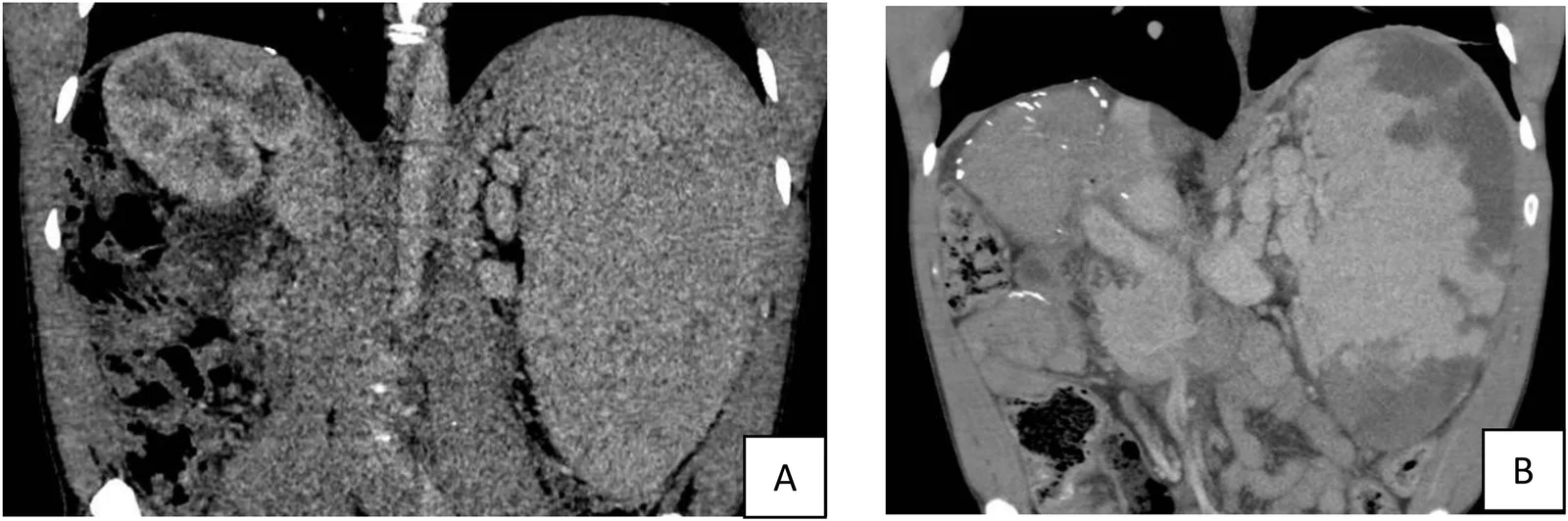
Coronal images showing pre-treatment splenomegaly (A) and changes following gel foam embolization treatment (B).

For these reasons, he underwent a combined transjugular biopsy and hepatic vein interrogation in addition to visceral arteriography; these procedures were performed in the same session due to his long travel time and social circumstances. At that time, there was no evidence of hepatic venous stenosis with a calculated hepatic sinusoidal pressure of 6 mm Hg, suggesting mild portal hypertension. The celiac arteriogram showed an occluded splenic artery with a tangled network of collaterals to the spleen via gastric arteries. The superior mesenteric arteriogram also revealed numerous collaterals from the IPDA to the massively enlarged spleen, and a proximal 1.8 cm junctional IPDA aneurysm. Further IPDA collateral interrogation with selective microcatheterization showed clear filling of the splenic artery confirmed on cone beam CT. Since venous outflow steno-occlusion was not the culprit for his portal hypertension, and he recently had a life-threatening variceal bleed, gel foam embolization was then performed from an IPDA collateral with a goal of 40–50% devascularization of the spleen.

The immediate initial post-procedure period was uneventful, and he was discharged the same day, but he developed post-embolization syndrome on post-procedure day 2. His nausea and abdominal pain were sufficiently managed at home with a combination of steroids, oral narcotics, and acetaminophen. Additionally, given his symptoms and desire for earlier re-evaluation, a computed tomography scan of the abdomen and pelvis (CTAP) with contrast was performed at week 2 and week 4 post-procedure. Both sets of imaging demonstrated 40% devascularization of the spleen and a repeat EGD by his OSH gastroenterologist showed decompressed esophageal varices.

However, the patient’s continued desire to weightlift with the junctional IPDA aneurysm resulted in a multidisciplinary decision to treat the aneurysm. In addition to the unpredictable pattern of growth and rupture associated with these types of lesions, there was additional concern that repeated bouts of increased blood pressure associated with the Valsalva maneuver (common while weightlifting) would further increase the risk of aneurysm rupture in this specific patient.

The patient returned for embolization of the aneurysm a few months after the original procedure. Selective catheterization was performed of the SMA, revealing the IPDA junctional aneurysm arising from a proximal branch with two outflows from the sac (ie, occurring at a branch point). A “front-door–back-door” approach, including both outflow vessels, was employed as detailed in Figures 2A–E and its accompanying legend. A combination of oversized microvascular plugs (MVPs), framing coils, and filling coils used to reduce blood flow within the aneurysm to stasis.

**Fig. 2:**
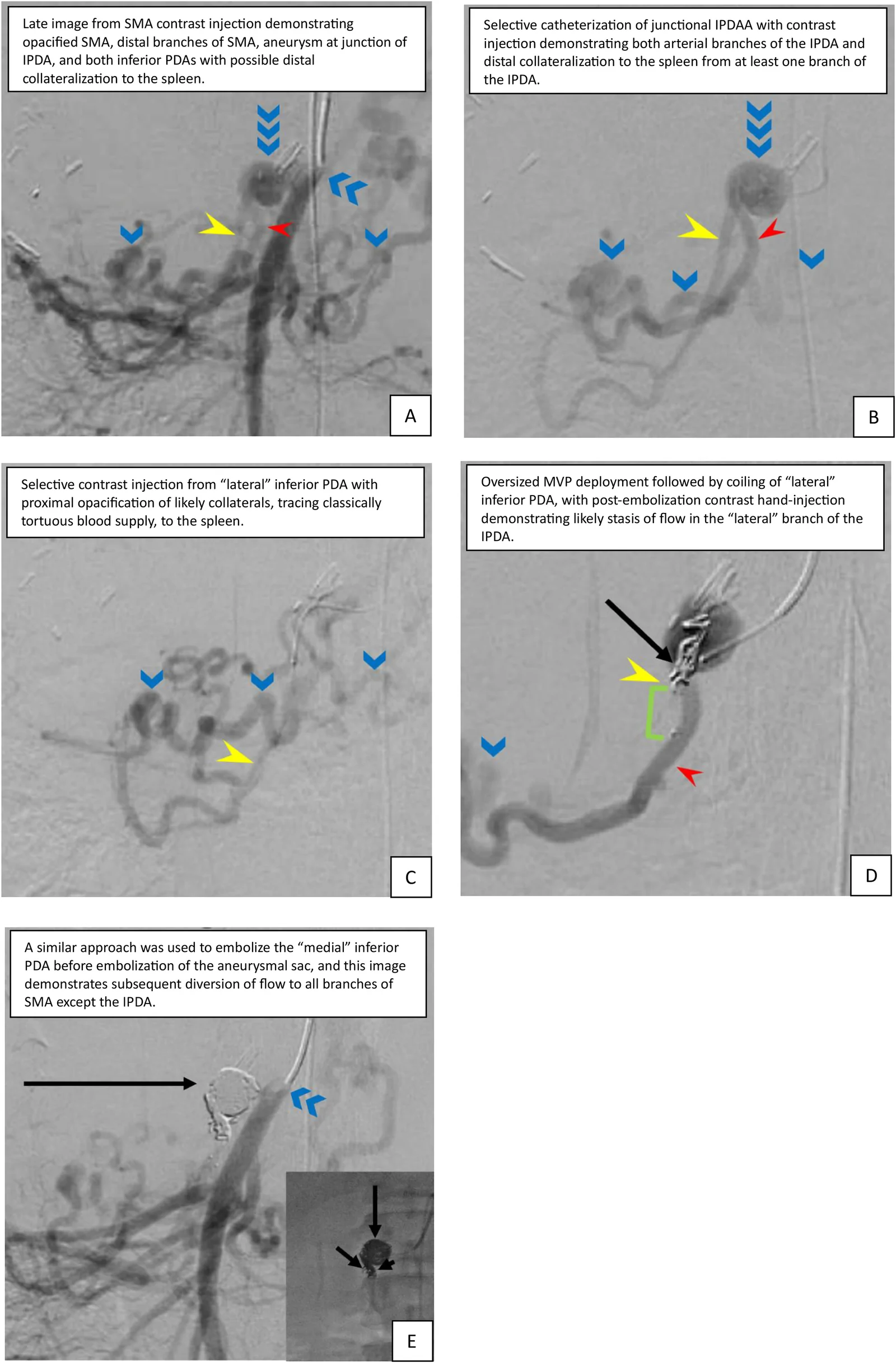
Blue single chevrons demarcate arterial supply to the spleen; blue double chevrons indicate superior mesenteric artery; blue triple chevron highlights the IPDA junctional aneurysm; small red arrow head calls attention to “medial” inferior PDA; large yellow arrow head points towards the “lateral” inferior PDA; large green bracket highlights deployed 5 mm MVP; small white bracket indicates 3 mm MVP; large black arrow points towards one deployed Ruby framing coil 12 mm × 40 cm, one deployed Ruby soft coil 8 mm × 60 cm, and two deployed Ruby soft coil 8 mm × 35 cm; medium black arrow points towards one deployed Ruby framing coil 4 mm × 10 cm; and small black arrow points towards one deployed Ruby framing coil 5 mm × 20 cm and one deployed Ruby framing coil 4 mm × 15 cm. Classically, there is only the anterior and posterior inferior PDA; "medial" and "lateral" is used as an alternative because the relative anterior-posterior positioning was not determined. Descriptive captions for subparts A-E are included on the figure itself.

Repeat CTAP was performed at 1 month and 4 months post-intervention and demonstrated splenic infarcts (Fig. 1B), with no further filling of the aneurysm. He returned to normal physical activity without issue. An IRB approval was not required for this case.

## Discussion

Atheroma formation alone was thought to be the predisposing insult that caused aneurysm formation in the single instance of a superior mesenteric aneurysm reported in 1932 [2]. Several decades later, in 1973 and 1978, Sutton [11] and Kadir [12] first postulated that celiac axis steno-occlusion—whether it be due to external compression, or internal occlusion, or intrinsic stenosis—led to compensatory flow through collateral vessels and could ultimately lead to aneurysm formation. However, only recently were the hemodynamics of the pancreaticoduodenal arcades in patients with PDAAs both quantitively and qualitatively studied [13]. Compared to healthy counterparts, patients with PDAAs had higher velocities, increased turbulent flow patterns, heterogenous wall shear stress distribution, and a higher oscillatory shear index [13].

Though this case diverges from the literature in its presentation and course, it reaffirms the suspected underlying pathophysiology and biomechanics involved in the formation of PDAAs. Ultimately, the increase in flow through a relatively narrow vessel remains the primary cause of eventual PDAA formation.

It remains unclear as to why the patient’s spleen continued to enlarge post-transplant in the absence of obvious venous outflow issues, a ligated splenic artery, and no evidence of rejection or cirrhosis on serial hepatic biopsies. A hematologic source was largely excluded after bone marrow samples were unremarkable. His primary endocrinologist also confirmed that his glycogen storage disease was non-contributory. He had Von Girke subtype a, not subtype b, and never required chronic filgrastim use— which can hypothetically lead to splenic sequestration of platelets/splenomegaly. The patient’s platelet count first demonstrated a decline from 219k/uL to 90k/uL a year after the transplant, and then eventually to 33k/uL over the next decade. As such, sequestration remains the most likely source of his thrombocytopenia and the driver of his engorged collateral arterial supply to the spleen. Regardless of the inciting cause, the patient developed massive splenomegaly and portal hypertension leading to bleeding esophageal varices.

This report describes a case of portal inflow reduction by partial splenic embolization via SMA collaterals which ultimately revealed an IPDA junctional aneurysm. This aneurysm was later treated and contributed further to reduction in portal inflow. As a result, the patient had an excellent response to therapy in the short-term, with de-pressurization of esophageal varices and no further episodes of bleeding; he was also able to resume weight-lifting without ill-effect, although it was strongly recommended that he avoid these activities. This case builds upon the literature of flow-related visceral aneurysms by confirming the underlying pathophysiology and the endovascular approaches to management despite a unique set of precipitating conditions.

## Patient consent

Complete written informed consent was obtained from the patient for the publication of this study and accompanying images.
